# Development of a support vector machine learning and smart phone Internet of Things-based architecture for real-time sleep apnea diagnosis

**DOI:** 10.1186/s12911-020-01329-1

**Published:** 2020-12-15

**Authors:** Bin Ma, Zhaolong Wu, Shengyu Li, Ryan Benton, Dongqi Li, Yulong Huang, Mohan Vamsi Kasukurthi, Jingwei Lin, Glen M. Borchert, Shaobo Tan, Gang Li, Meihong Yang, Jingshan Huang

**Affiliations:** 1 Shandong Provincial Key Laboratory of Computer Networks, Qilu University of Technology (Shandong Academy of Science), Jinan, China; 2 School of Computing, University of South Alabama, Mobile, AL 36688 USA; 3 College of Allied Health Professions, University of South Alabama, Mobile, AL 36608 USA; 4 Ocean School, Fuzhou University, Fuzhou, China; 5 College of Medicine, University of South Alabama, Mobile, AL 36688 USA

**Keywords:** Sleep apnea, Obstructive sleep apnea syndrome, SpO2, Support vector machine

## Abstract

**Background:**

The breathing disorder obstructive sleep apnea syndrome (OSAS) only occurs while asleep. While polysomnography (PSG) represents the premiere standard for diagnosing OSAS, it is quite costly, complicated to use, and carries a significant delay between testing and diagnosis.

**Methods:**

This work describes a novel architecture and algorithm designed to efficiently diagnose OSAS via the use of smart phones. In our algorithm, features are extracted from the data, specifically blood oxygen saturation as represented by SpO2. These features are used by a support vector machine (SVM) based strategy to create a classification model. The resultant SVM classification model can then be employed to diagnose OSAS. To allow remote diagnosis, we have combined a simple monitoring system with our algorithm. The system allows physiological data to be obtained from a smart phone, the data to be uploaded to the cloud for processing, and finally population of a diagnostic report sent back to the smart phone in real-time.

**Results:**

Our initial evaluation of this algorithm utilizing actual patient data finds its sensitivity, accuracy, and specificity to be 87.6%, 90.2%, and 94.1%, respectively.

**Discussion:**

Our architecture can monitor human physiological readings in real time and give early warning of abnormal physiological parameters. Moreover, after our evaluation, we find 5G technology offers higher bandwidth with lower delays ensuring more effective monitoring. In addition, we evaluate our algorithm utilizing real-world data; the proposed approach has high accuracy, sensitivity, and specific, demonstrating that our approach is very promising.

**Conclusions:**

Experimental results on the apnea data in University College Dublin (UCD) Database have proven the efficiency and effectiveness of our methodology. This work is a pilot project and still under development. There is no clinical validation and no support. In addition, the Internet of Things (IoT) architecture enables real-time monitoring of human physiological parameters, combined with diagnostic algorithms to provide early warning of abnormal data.

## Background

People in high intensity positions generally develop more sleep disorders [1]. Furthermore, sleep disorders often result in additional health problems since sleep is essential for our overall mental and physical health. Sleep apnea syndrome (SAS) is a wide spread, common sleep disorder affecting over 4% of men and 2% of women worldwide [2]. Notably, only 20% of people with SAS are actually aware they present this disorder [3].

The current tool for diagnosing SAS is polysomnography (PSG). PSG monitors breathing airflow [4], breathing events [5], snoring [6], blood oxygen saturation (SpO2) [7], electrooculography (EOG) [8], electroencephalography (EEG) [9], and electrocardiography (ECG) [10]. While effective in diagnosis, PSG carries several limitations. First, PSG is quite labor intensive as it requires a specific series of recording systems [11]. Second, PSG requires continual, hands-on supervision as patients must wear numerous body sensors. And third, PSG equipment carry significant costs with the core PSG Machines costing ~ $6000 [12–14].

Furthermore, the construction of a diagnosis model for SAS is non-trivial. The default employment of all features generally results in less than desirable performance, due to several features typically being irrelevant and/or redundant; additionally, poor feature selection can similarly also result in suboptimal performance. As such, finding an optimal set of features, without undue burden on the analysis, is essential.

SpO2 and ECG are the two features most widely studied in physiological signals collected by PSG. SpO2 represents the percentage of hemoglobin in the blood. In hypopneas status, the reduction of airflow causes a reduction in oxygen saturation. Typically, classifiers employ time frequency domain and time domain SpO2 features for apnea detection, and this strategy is the most widely accepted in the field [5]. However, there are a number of issues with existing solutions; they require the use of expensive equipment to collect the day and/or they require some offline processing in order to perform a diagnosis.

In order to detect apnea, a pulse oximeter, which is a kind of portable sensor, is utilized to record SpO2. The use of pulse oximeters carries several advantages. First, pulse oximeters produce clearer record details than ECGs for evaluating hypopnea status [6]. Second, a regular pulse oximeter costs ~ $100 dollars, which is dramatically less expensive than a PSG machine. Third, combined with our proposed classification method and our smart phone-based system, it allows both on-line diagnosis and addresses many PSG-based issues with diagnosing obstructive sleep apnea syndrome (OSAS) (e.g. labor costs, computing resources, and time).

Internet of Things (IoT) strategies are widely utilized in an array of areas including sensor related applications [15–17] and detection systems [18–20]. Furthermore, combining IoT, cloud computing, and machine learning has previously proven effective for precise, real-time sleep apnea detection and diagnosis [21]. Cloud computing reduces the cost on servers, hardware, software licenses, and safety maintenance [22]. For instance, a sleep apnea monitoring system was proposed by Bsoul et al. [23]. The system, which utilized ECG readings, allowed for real-time monitoring; however, the diagnosis requires the processing to be done on the mobile phones, which could be problematic.

Recently, major research efforts in sleep apnea detection have focused on reducing costs through automation. One major focus of automation has been the use of preprocessing algorithms, which can be carried out on acquiring both testing and training data to improve diagnostic model accuracy and to reduce the complexity of the classification algorithms as well. The monitoring and diagnosis of SAS often include three major stages: feature extraction, feature selection, and pattern classification [24]. Publicly available physical databases are widely utilized for evaluating the efficiency of preprocessing operations [25–28].

In terms of prediction, Oliver and Flores-Mangas developed a detection algorithm in oximetry [29] that operated in real-time; however, they did not provide a comparison to standard PSG methodologies in terms of performance. Another approach, implemented by Heneghan et al. [30], used linear discriminative analysis to classify apnea using eight ECG and five SpO2 features. The sensitivity and specificity of their classifications were 51.4% and 87.3%, respectively; they noted that if either the ECG or SpO2 feature sets were missing, their model could still work, although at reduced performance. McNames and Fraser [25] and Raymond et al. [26] both achieved accuracies of over 90% based on one-minute basis and one-minute segment analyses of the Apnea-ECG Database [31]. Notably, however, both methods required manual activities. McNames and Fraser used manual inspections to identify apneic episodes [25], while Raymond et al. relied on manual editing to improve performance [26].

Machine learning has been applied to many areas, such as medical diagnosis [32] and fault detection [33]. Importantly, unlike previously developed algorithms, we propose a Support Vector Machine (SVM) based, IoT framework embedded scheme for diagnosing sleep apnea. SVM methods have become a standard means for data classification primarily due to their simplistic numerical comparisons and optimal solution determination ease for a specific context [25]. It has been extensively used in biomedical signal classification applications, such as electromyography (EMG) [34], electroencephalogram (EEG) [35], and electrocardiogram (ECG) [36]. In addition, several approaches have also recently been developed for determining the optimal hyperplane of kernel-based, non-linear SVMs [37] in which a hyperplane is constructed that separates data representing two different groups (classes).

In our algorithm, the values of SpO2 are divided into one-minute segments as a main subset of characteristic data, and the characteristics, including maximum, minimum and other features (shown in Table 1) of this SpO2 segment are utilized to diagnose apnea. From the aspect of IoT framework for monitoring and diagnosing sleep apnea, researchers have developed some frameworks. Rofouei et al. [38] presented a non-invasive, wearable neck-cuff IoT system for monitoring the sleep of people. Kumar et al. [39] proposed a framework to help patients with OSA. The framework used sensors within the patient environment as well as wearable devices to monitor and collected a variety of data. Data collected included, but was not limited to, room temperature, room humidity, patient blood pressure, heart rate, body temperature, and blood oxygenation. The collected data by the sensors were sent to the cloud layer via a mobile phone or similar device; the data then could be used for analysis. However, there is a lack of automatic diagnosis in current studies. Thus, we combine the IoT framework based sleep monitoring system with our algorithm for automatic diagnosis and remote warning. Finally, our utilization and preprocessing of SpO2 data for diagnosis improves the accuracy of detection and reduces the false positive.

**Table 1:** SpO2 statistical features

Feature Name	Description
*S*_*max*_	Maximum SpO2 in 1 min
*S*_*min*_	Minimum SpO2 in 1 min
*S*_*mean*_	Average SpO2 in 1 min
*S*_*vari*_	SpO2 Variance in 1 min
*CorreC*	Correlation coefficient of 1 min SpO2 sample data
*ZCount*	The count of the number of zero crossing points within a segment where *S*_*mean*_ is the baseline
*SpSlope*	Slope of the regression line in 1 min
*AbSlope*	Absolute slope
*Bias*	Bias of the regression line of SpO2 in 1 min
*Dmean*	Delta index: 1 min average of the absolute differences between two successive mean values of SpO2 signal over 12 s intervals

This article is a significant extension of our previous work [40] with all sections markedly extended in this report. Substantial extensions (excluding minor modifications) include: (1) Background: expanded the introduction of PSG along with six additional references; added more explanations on SpO2 and oximeter; added more explanations on IoT along with four references; added more elaborations on recent research on sleep apnea monitor and detection along with nine references; added more about the application of machine learning along with two references. (2) Materials and Methods: added four citations to describe SVM; expanded the description of the SVM models, especially the detailed explanation of the ten features used in SVM; added more details of our IoT architecture including the introduction of three main components. (3) Results and Discussion: added more explanations of sensitivity, specificity, and accuracy; added a table to show the True Negative (TN), True Positive (TP), False Negative (FN), False Positive (FP); and False added more detailed explanations of the structure of our data, along with three figures containing participant amounts categorized by age, Body Mass Indexes (BMIs), and Apnea Hypopnea Indexes (AHIs); compared our algorithm with Deep Belief Network (DBN) and Adaptive Boosting (AdaBoost) using the same data features along with one table and one figure to show the better performance of our algorithm; simulated and compared the packet loss and packet retransmission of both 4G and 5G network along with two figures.

The most significant innovations in this work are the IoT architecture and the diagnosis algorithm of sleep apnea based on SVM. Importantly, we proposed to use the SpO2 as the feature data for training and testing on SVM, DBN and AdaBoost respectively. Therefore, the best algorithm is selected as the diagnosis algorithm of sleep apnea.

The rest of this article is structured as follows. The Methods section introduces SVM, presents our IoT architecture, and describes our algorithm. The Results section reports experimental findings, and the Discussion section discusses our results and concludes with proposed future directions.

## Methods

**Fig. 1:**
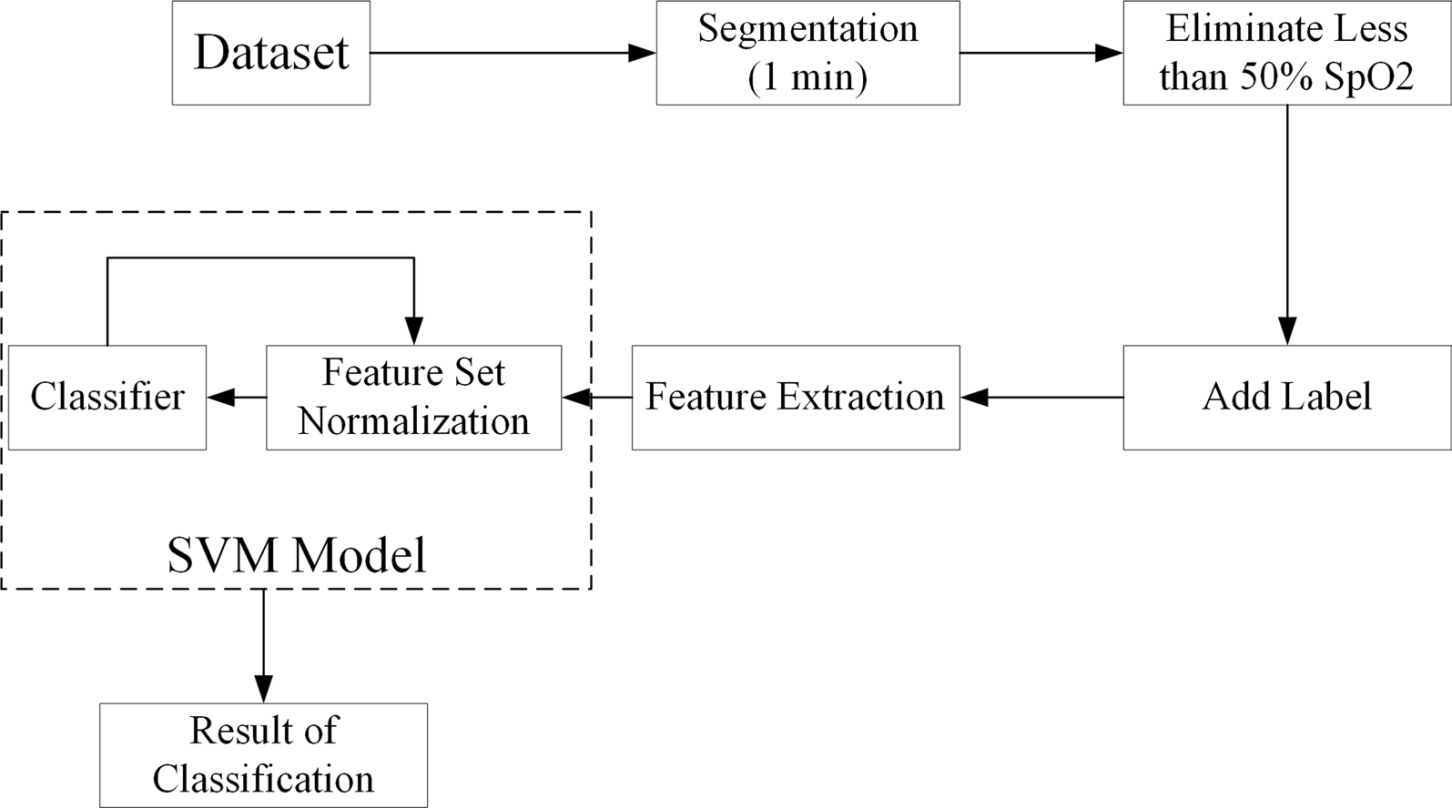
Process of our method

In this study, we utilized data curated by St. Vincent’s University Hospital at University College Dublin; we entitle this Sleep Apnea Database as the UCD Database [41]. SpO2 features were obtained from the database after pre-processing and then a SVM is used for diagnosing SAS. The general process is depicted in Fig. 1.

### Purpose and overview of our method

The method presented in this work provides a novel means of detecting likely apnea events through analyzing SpO2 data. To achieve this, SpO2 data are preprocessed and divided into single minute intervals (segments). Each segment is then classified as either an apnea event, which is defined as an at least ten second cessation of breathing [42] or as an non-apnea event. Smaller interval segments (e.g. 30 s) were not employed due to the increased likelihood of interrupting an apnea event. As such, 1 min intervals were selected to label data in this study. We utilize MATLAB R2014b for preprocessing and find this preprocessing step reduces complexity and greatly enhances overall performance.

In addition, SpO2 signals are individually down sampled to one Hertz (Hz). SpO2 values < 50 are regarded as noise [43] (as recommended by domain experts). Next, ten features are computed from each segment which is showed in Table 1. To start, the SpO2 maximum, minimum, variance, mean, and correlation coefficient are computed; these are correspondingly designed as *S*_*max*_, *S*_*min*_, *S*_*vari*_, *S*_*mean*_, and *CorreC*. *S*_*mean*_, for each segment, is used as a baseline to calculate *ZCount*, which is the number of times a zero crossing occurs. In addition, each segment employs linear regression to construct a representative line from which three features: the regression line slope (*SpSlope*), the bias (*Bias*), and the absolute value (*AbSlope*). Finally, a delta index, *Dmean* is used as another Sleep Apnea–Hypopnea Syndrome (SAHS) detecting parameter; this requires two steps. The first step requires that the mean of SpO2 signal be calculated for every 12 s interval within a segment. The second step requires that the absolute differences between consecutive mean within the segment be averaged; this is the *Dmean*. Once all the features for each segment is calculated, a classifier can be constructed.

Our SVM classifier, which uses the Radial Basis Function (RBF) kernel, requires two principal user-provided parameter settings. The two user-provided parameters are *C*, which is set to 1,000, and σ, which is set to five. In our experiment, they are set by using trial and error. In addition, every feature is scaled to the range of 0–1 because SVMs, in general, are sensitive to feature dynamic ranges. We use an freely available Python package scikit-learn (version 0.19.0) to create the SVM and to test it; it is executed in the cloud in order to evaluate feature set performance with ten-fold cross-validation being used to train and then validate our methods.

### IoT system architecture

**Fig. 2:**
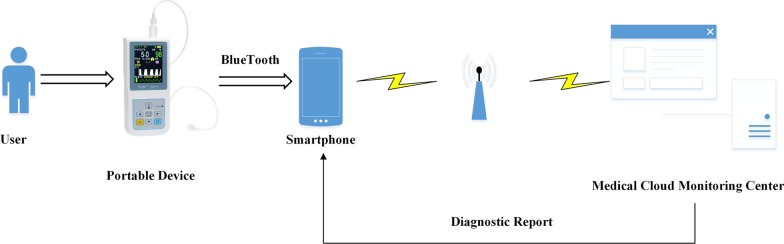
IoT system architecture

We now propose a sleep apnea diagnosing scheme using the framework of the IoT (Fig. 2). This scheme is comprised of three primary components: a smart phone monitoring system, a portable device, and a medical cloud monitoring center. SpO2 data are read by the portable device from the human body surface, which is then transmitted to a mobile phone via a low-power consumption Bluetooth module. After data are uploaded, our smart phone monitoring system preprocesses physiological data then uploads this data into a medical cloud-based monitoring center which carries out three tasks: long-term patient data storage, analysis, and visualization. It also provides relevant analysis to help medical staff with their diagnoses allowing medical staff to more efficiently provide effective treatment for patients with sleep apnea.

1. *Portable device design* As SpO2 signals must be directly collected for data analysis, a portable medical oximeter was used to monitor SpO2. Using a portable medical oximeter brought us the following benefits. First, this device is simple to operate and convenient to wear. The patient only needs to wear a fingertip blood oxygen probe. Second, the device is seamlessly integrated with four functional modules: (a) single chip microcomputer module, (b) multiple sensors, signal processing module, (c) power management module and (d) wireless communication module. And also, it can measure SpO2, breathing rate, and pulse rate in real-time while the patient is sleeping.
2. *Smart phone monitoring system* We also designed an application to help monitor the status of the patient. The application was implemented in Java using the Android Studio 3.0 environment. It was debugged on a smart phone (Huawei version 10) using the Android operating system (version 8.0). The two interfaces of the application correspond to two functions. The first function is to monitor and display the history data including the waveform and digital form of physiological signal. Another function is to provide users with real-time diagnose. Note that in the backend, the smart phone handles the storage, upload, and download of data. Also note that the application realizes data acquisition and preprocessing, including format conversion and related calculation.
3. *Cloud monitoring center* Specifically, the center manages the following tasks: (a) storage, (b) analysis, and (c) visualization. By using the center, the following advantages are obtained. First, in terms of database scalability, the platform allows multiple medical devices to simultaneously connect and ensures the stability and reliability of two-way communication between devices and the cloud. Second, it provides different network device access schemes such as 2G, 3G, 4G, 5G and Wi-Fi, as well as device side Software Development Kit (SDK) of Message Queuing Telemetry Transport (MQTT), Constrained Application Protocol (CoAP), Hypertext Transfer Protocol (HTTP)/Hypertext Transfer Protocol Secure (HTTPS) and other protocols. The ability to handle multiple network schemes and protocols not only meets the real-time demand of long connection, but also meets the low power demand of short connection. Finally, in terms of security, it supports the custom data symmetric encryption channel on Transmission Control Protocol (TCP)/User Datagram Protocol (UDP), which is suitable for power-sensitive and resource-constrained medical devices.

### Support vector machine learning

SVM learning is a widely utilized method of binary classification [44]. To create the model, an SVM exploits the information found within the training set. Once created, the model is later used to classify individual instances within the test data. During training, an SVM identifies hyperplane that best separates two classes:

$$w^{T} X_{i} + w_{0} = 0$$

In particular, an SVM assumes the “best” hyperplane is a maximum margin hyperplane, which can be treated as an optimization problem. Given a training dataset $X = (X_{1} ,X_{2} , \ldots ,X_{T} )$, with associated class $y_{i} \in \left\{ { - 1,1} \right\}$, 1 for normal and − 1 for abnormal, for $i \in (1, \ldots ,T)$, a hyperplane is obtained through the following mathematical constraints:

$$\begin{array}{*{20}l} {\mathop {{\text{Minimize}}}\limits_{{{\text{w,w}}_{{0}} }} } \hfill & {\frac{1}{2}\left\| w \right\|^{2} + C\sum\limits_{i = 1}^{m} {\xi_{i} } } \hfill \\ {\text{Subject to}} \hfill & {w^{T} X_{i} + w_{0} \ge 1 - \xi_{i} \; \, if\;X_{i} \in y_{1} } \hfill \\ {} \hfill & {w^{T} X_{i} + w_{0} \ge - 1 + \xi_{i} \;if\;X_{i} \in y_{2} } \hfill \\ {} \hfill & {\xi \ge 0} \hfill \\ \end{array}$$

Margin distance equals ${2 \mathord{\left/ {\vphantom {2 {\left\| w \right\|}}} \right. \kern-\nulldelimiterspace} {\left\| w \right\|}}$, and structural risk minimization involves a slack variable $\xi_{i}$ to be introduced in order to reduce overfitting while *C* is a user-defined invariant. The nearest coordinate in the training data is used to calculate the distance to the hyperplane. The larger the distance, the better the classification effect with these specific data points referred to as support vectors that satisfy the rule:

$$y_{i} (w^{T} \cdot X_{i} + w_{0} ) = 1$$

where $w$ represents the hyperplane’s normal vector, and $w_{0}$ hyperplane’s function bias of the. After which, normal vector $w$ can be calculated using the training dataset:

$$w = \sum\limits_{i = 1}^{n} {\alpha_{i} y_{i} X_{i} }$$

where $\alpha_{i}$ is the optimizing Lagrange multiplier. If $\alpha_{i}$ does not equal zero, a set of sample coordinates represent a support vector. Notably, $\alpha_{i}$ equaling zero will not disrupt the training model. In addition, the classification of *X*_*i*_ is based on the function *h*(*X*_*i*_):

$$y_{i} = sign(h(X_{i} )) = sign(w^{T} \cdot X_{i} + w_{0} )$$

## Results and discussion

We have evaluated the specificity, sensitivity, and accuracy of our SVM classifier in terms of diagnosing SAS. We have also analyzed efficiency of the system when built upon IoT structure. The experimental environment employed was MATLAB R2014b, Intel (R) Core (TM) i7-6700 CPU @ 3.40 GHz.

### Metrics

We have used sensitivity, specificity, and accuracy as metrics to evaluate classification performance. They are defined as follows:

$$Sensitivity = \frac{TP}{{TP + FN}}$$

$$Specificity = \frac{TN}{{TN + FP}}$$

$$Accuary = \frac{TP + TN}{{P + N}}$$

where TP and TN represent the number of detected apneic segments and normal segments, respectively. FN and FP stand for the number of misidentified normal and apneic segments, respectively. Negative (N) and Positive (P) are the number of segments without and with apneic events. Sensitivity refers to the percentage of correctly classified apnea epochs, specificity as normal epoch percentage correctly classified, and accuracy as percentage of total segments correctly classified.

### Datasets

To evaluate our algorithm, we, as mentioned earlier, utilized the UCD Database that contains overnight, Jaeger-Toennies system-collected polysomnograms (in their entirety) obtained from 25 participants believed to exhibit disordered breathing during their sleep [41]. The Jaeger-Toennies system monitors all sleep related data including EEG, EOG, EMG, ECG, SpO2, etc. In our experiment, we only utilize the SpO2 data of these 25 participants. All participants were adults that consented to have their SpO2 monitored by fingertip pulse oximetry. The participant physiological properties are shown in Table 2.

**Table 2:** Physiological properties of subjects in UCD database

	Mean value	Range
Age (years)	50 ± 10	28–68
Body Mass Index (kg/m^2^)	31.6 ± 4	25.1–42.5
AHI	24.1 ± 20.3	1.7–90.9

**Fig. 3:**
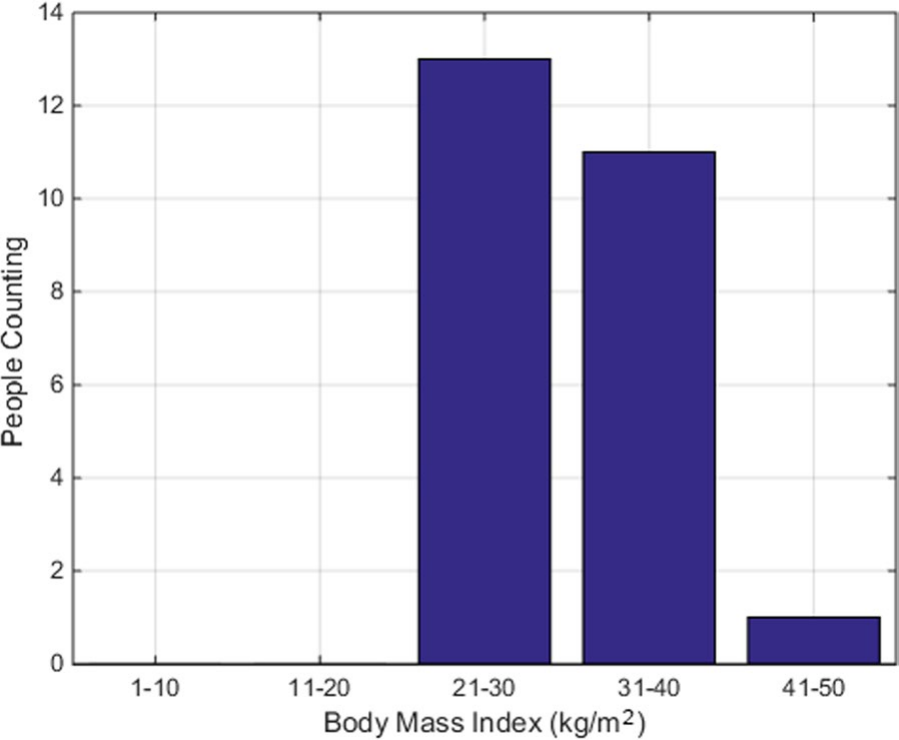
Age distribution of the 25 participants

**Fig. 4:**
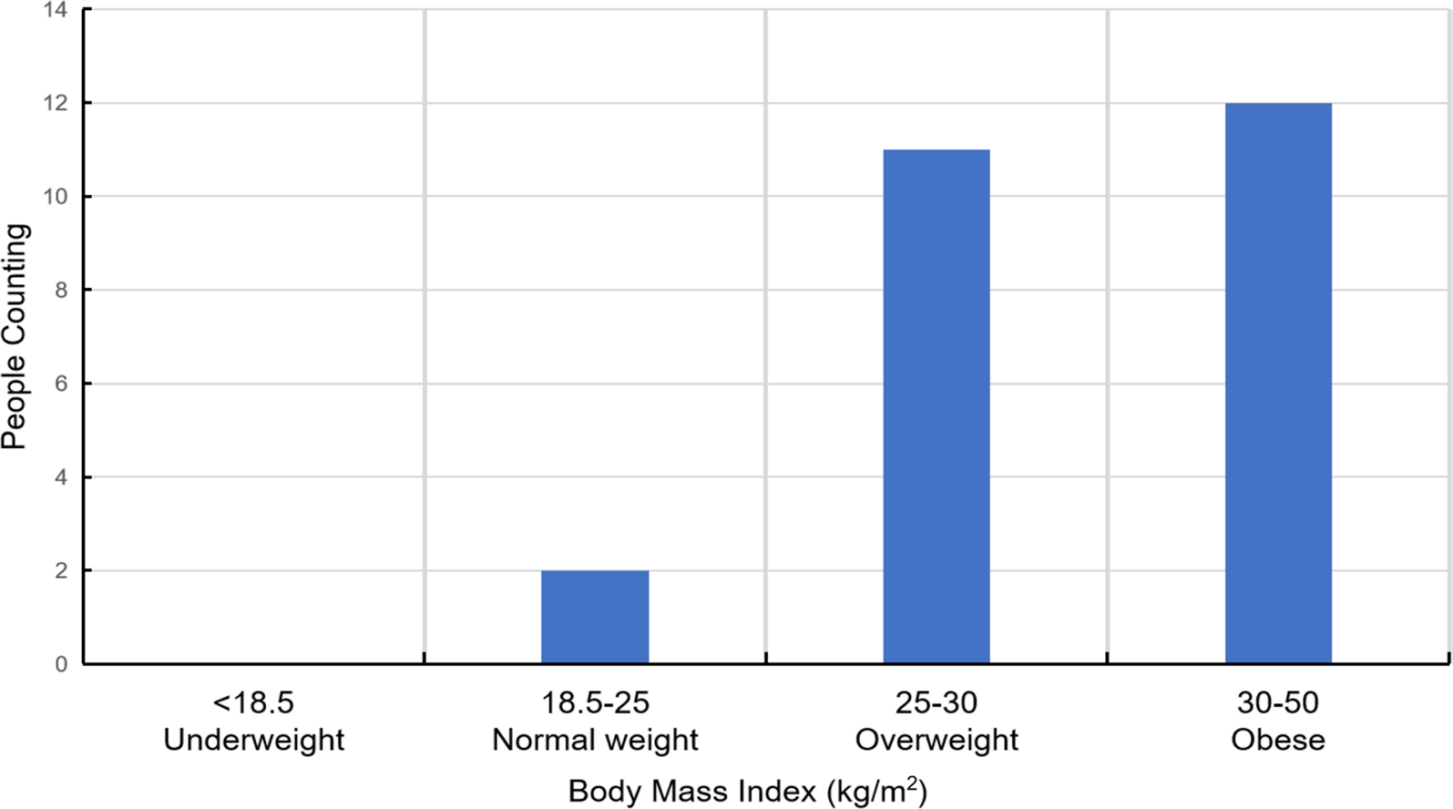
BMI distribution of the 25 participants

**Fig. 5:**
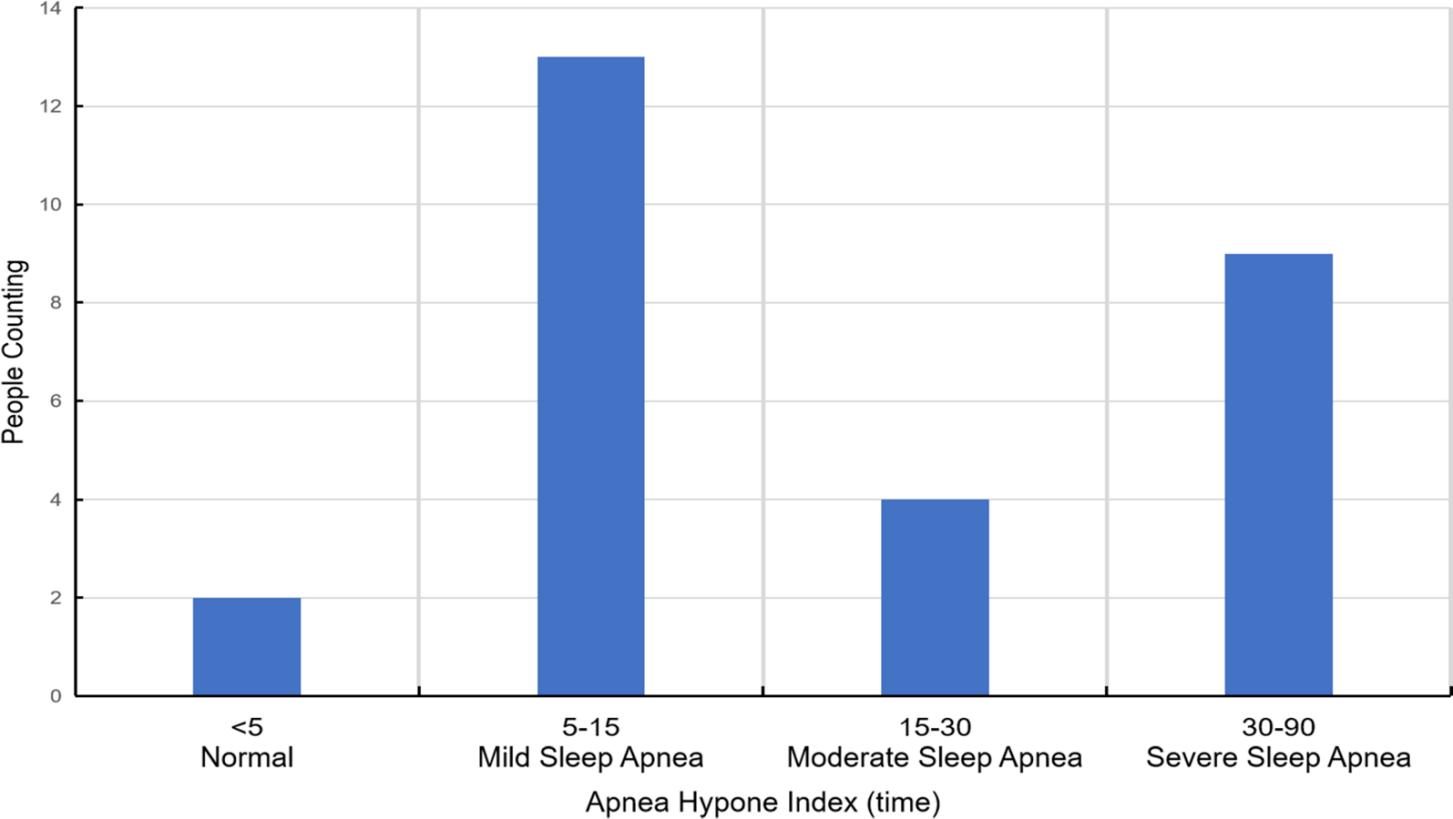
AHI distribution of the 25 participants

There were 21 males and four females with ages ranging from 28 to 68. As shown in Fig. 3, we find people with 37–54 years of age typically had more SAS. Similarly, the BMIs range from 25.1 to 42.5 kg/m^2^, the majority of which seem to have high BMIs. While high BMIs appear to associate with a high risk of sleep apnea, this could be just co-incidence based on the data distribution. Details of the BMI are provided in Fig. 4. Moreover, patient AHIs range from 1.7 to 90.9 as shown in Fig. 5. In addition, all diagnostic information was annotated by experts to facilitate the classification of training data. With the help of domain experts, we treat every minute as a segment and assign each segment with a label, which is apnea or non-apnea.

In our experiment, we used the ten statistical features of SpO2 in order to learn and train. It allows us to verify that these features of SpO2 data can be used for the diagnosis of SAS. In all, the dataset has 1457 apnea events and 2278 non-apnea events.

### Performance comparison between DBN and AdaBoost

**Fig. 6:**
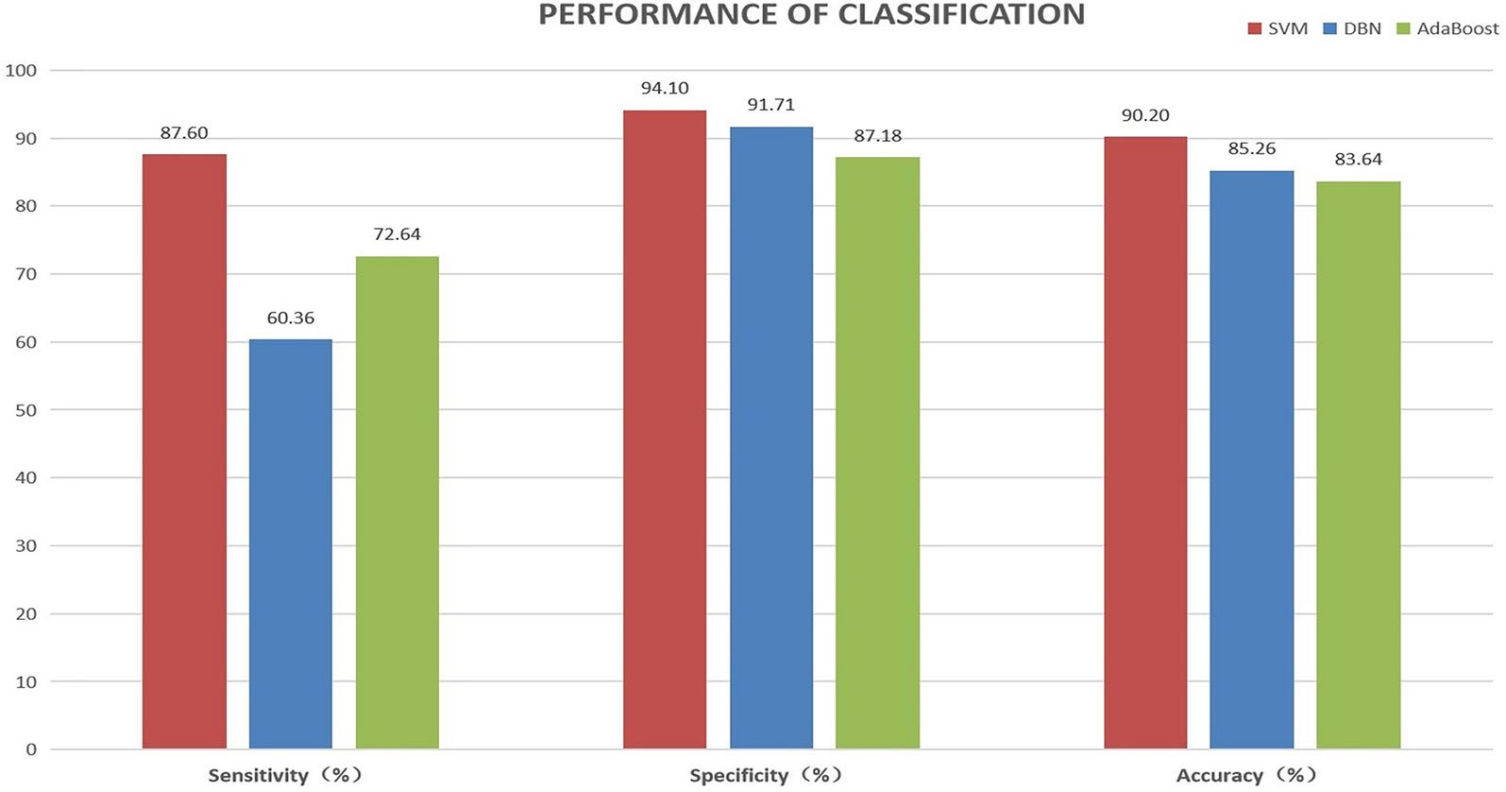
Performance comparison of SVM, DBM, and AdaBoost

We also tried DBN and AdaBoost using the same data set and features. Table 3 and Fig. 6 show the performance of the classification. We tried the DBN by using the default setting of it in TensorFlow (version 1.6), an open-sourced machine learning package. We used scikit-learn (version 0.19.0) to try AdaBoost. By using trial and error, the number of weak classifiers of AdaBoost was set to 10, 20, 30, and 40. Among them, 30 weak classifiers obtained the best experimental results. The performance using 30 weak classifiers was used to compare with our algorithm. The sensitivity, specificity, and accuracy of our SVM algorithm is 87.6%, 94.1%, and 90.2%, separately, which all surpass the DBN and AdaBoost. Based on sensitivity, specificity, and accuracy, which are common metrics to evaluate algorithm performance, it appears that the SVM algorithm is more suitable to diagnose SAS than deep learning algorithm like DBN and AdaBoost. With respect to DBN, we believe part of the reason the SVM performs better is that deep learning networks typically require large amount of data; the problem domain used is rather small, in terms of instances, for deep learning. It also should be noted we used the default parameters for DBN; tuning DBNs tends to be time-consuming compared to SVMs. With respect to AdaBoost, the use of multiple weak classifiers does not guarantee an optimal solution. In case, it can be the tree approach of divide and conquer does not result in the best approximations for the problem; it can also be that the default parameters for the AdaBoost algorithm could be better optimized. That said, Radial Basis SVMs tend to be a good choice for many problems, giving good results.

**Table 3:** Performance comparison of SVM, DBM, and AdaBoost

Classifier	% Sensitivity	% Specificity	% Accuracy
SVM	87.6	94.1	90.2
DBN	60.36	91.71	85.26
AdaBoost	72.64	87.18	83.64

Table 3 corresponds to Fig. 6

### 5G and 4G network simulation result

**Fig. 7:**
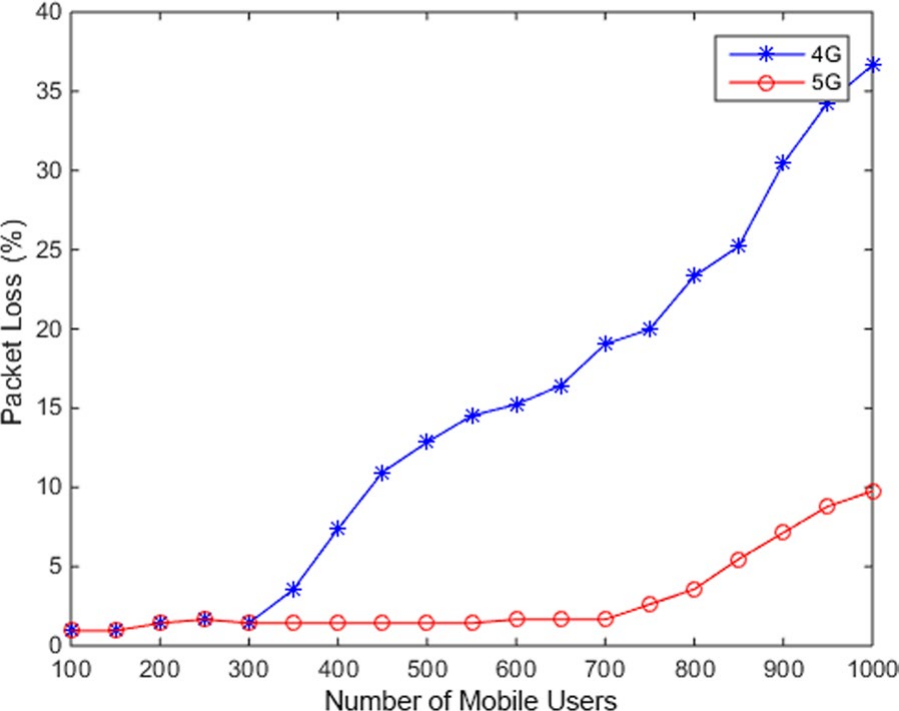
Packet loss rate in 4G and 5G Network

**Fig. 8:**
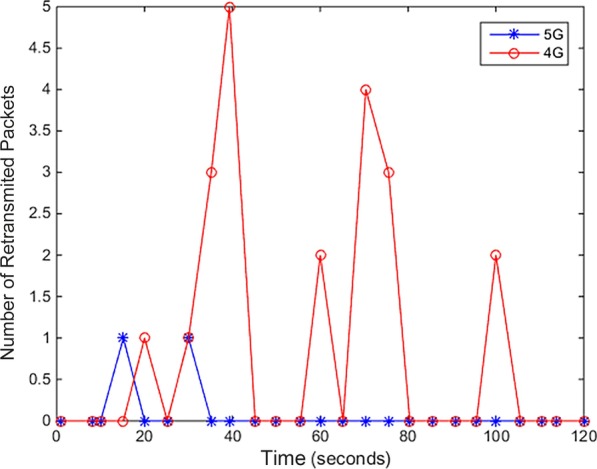
Packet retransmission in 4G and 5G

To evaluate the performances of different networks when user numbers increase, we simulated the data transmission between the server and the client using 4G and 5G network. In the 4G network simulation, we set the bandwidth, delay variation, and packet loss rate to 20 Mbps, two to forty milliseconds, and 0.1%, separately. In the 5G network simulation, we set the bandwidth, delay variation, and packet loss rate to 1000 Mbps, one to five milliseconds, and 0.1%, separately. The packet loss comparison result is shown in Fig. 7. It shows that the range of 5G packet loss is lower than the range of 4G packet loss. We also simulated the number of packets retransmitted when a mobile user transmits data in 4G and 5G Network. Results are shown in Fig. 8. As shown in the figure, the number of packets retransmitted in 4G network is higher than that in 5G network. Therefore, the 5G technology can effectively provide much larger data capabilities, and lower delays guarantee bandwidth availability for all users. We recommend users to use 5G network when using our application to ensure the transmission of data. Moreover, next generation 5G mobile networks are better able to provide IoT service and cloud computing service in streaming applications to mobile users and guarantee a higher Quality of Service (QoS) with significantly increased bandwidth.

### Apnea versus non-apnea

**Fig. 9:**
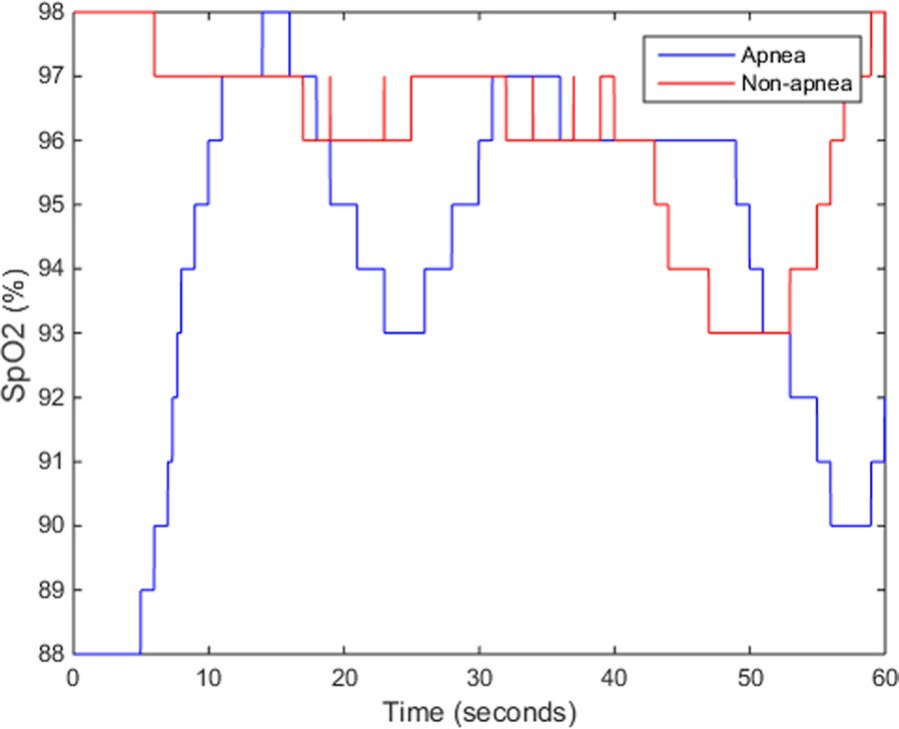
1 min of SpO2 data from both a normal and an OSAS patient

**Fig. 10:**
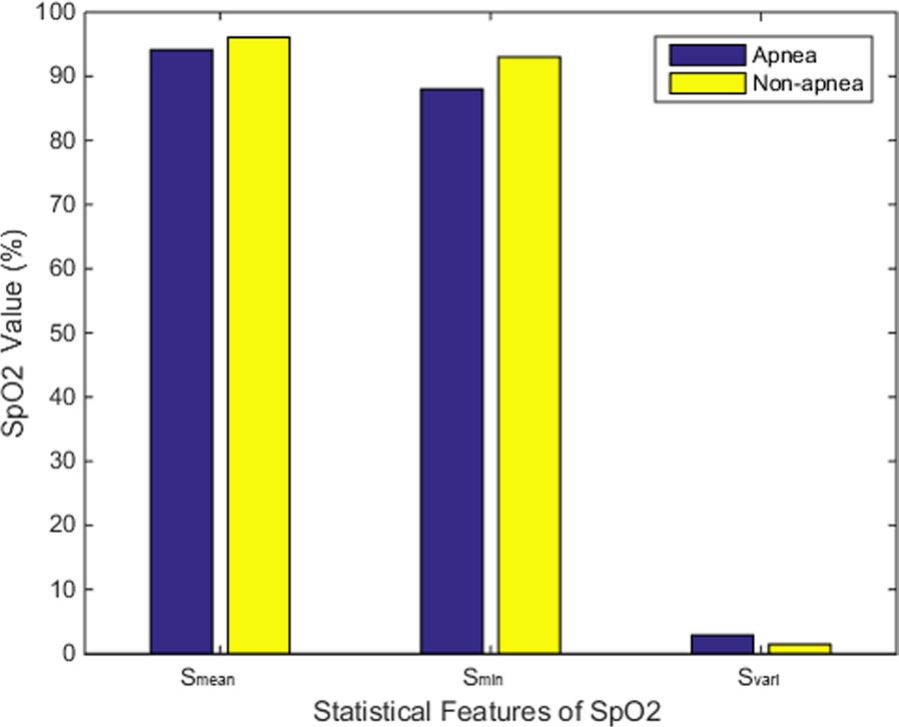
SpO2 mean, minimum, and variance statistical features calculated from SpO2 data obtained from normal and OSAS patients

An illustration of the normal and apnea conditions is shown in Fig. 9. As can be seen, apnea has a larger effect on oxygen saturation in the blood, which significantly impacts SpO2 levels; the minimum, mean, and variance statistical features derived from SpO2 for apnea and normal patients are shown in Fig. 10. In addition, our analysis clearly indicates our algorithm can accurately recognize apnea events occurring during sleep using the information derived from SpO2 data.

### The system test

**Fig. 11:**
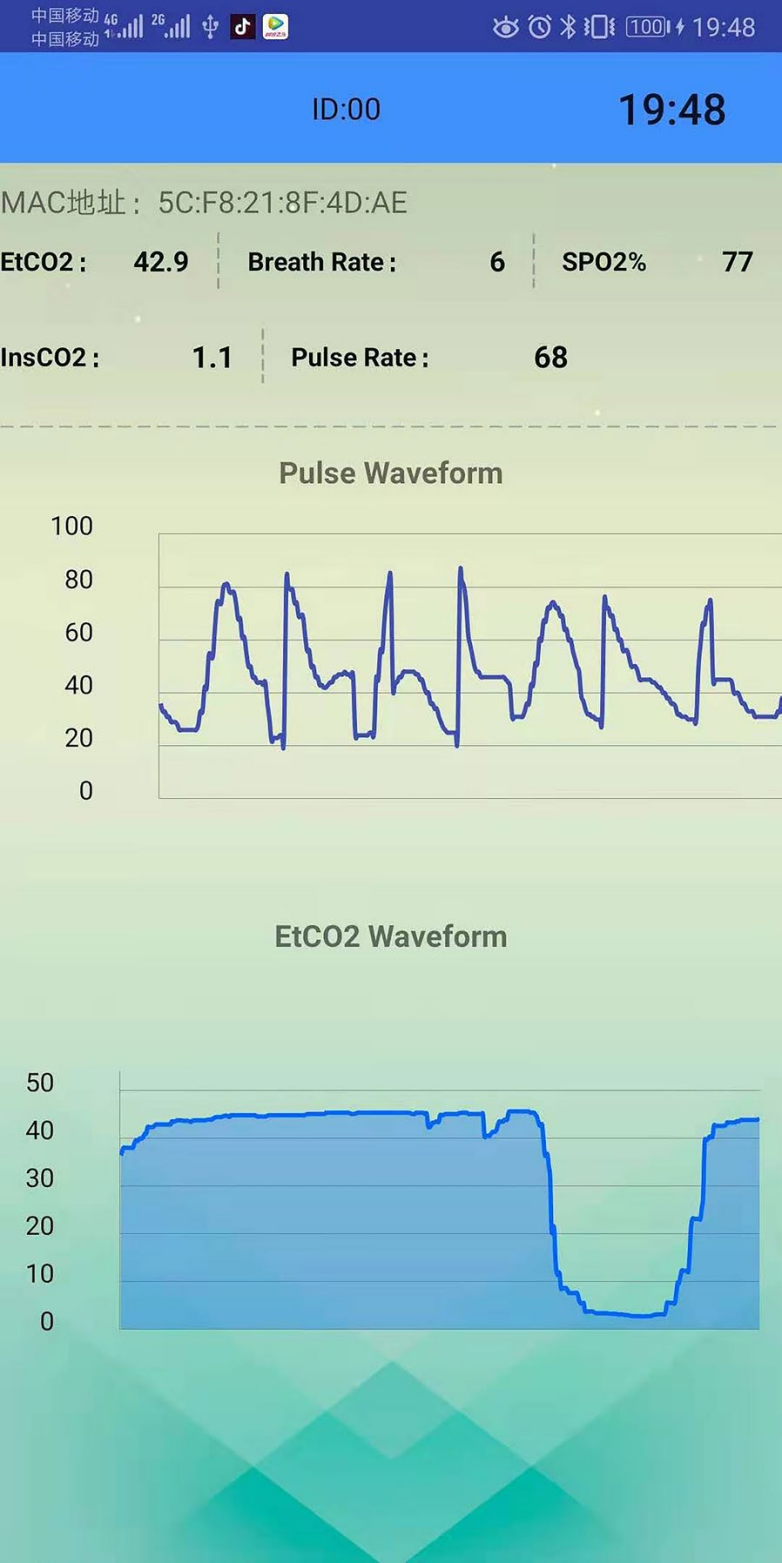
Physiological parameters in real-time

**Fig. 12:**
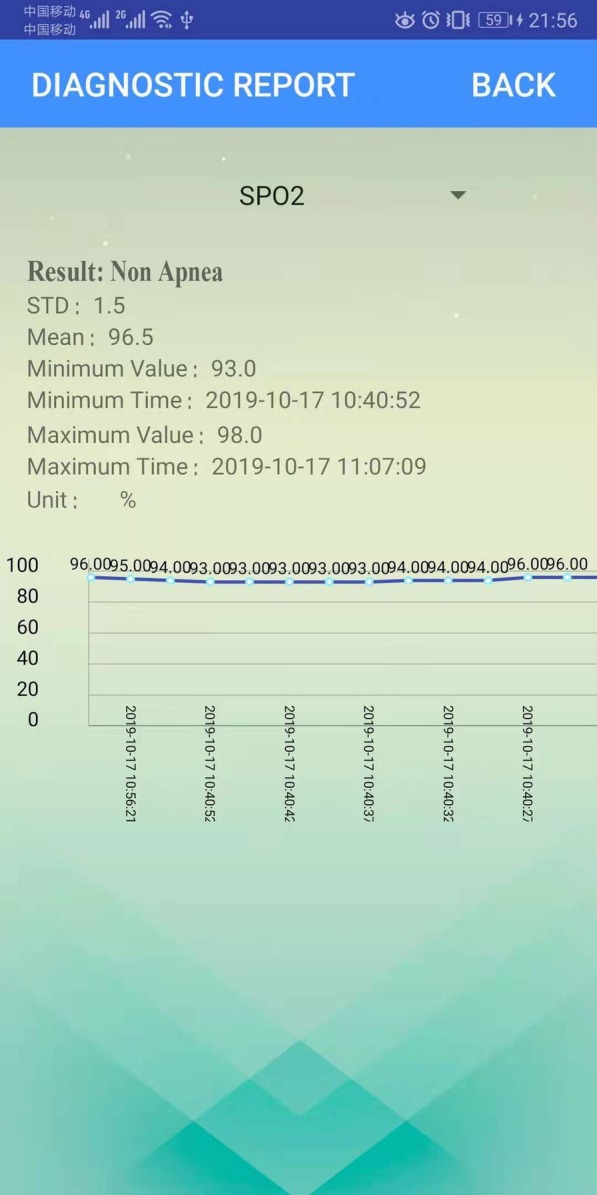
Non-apnea report

**Fig. 13:**
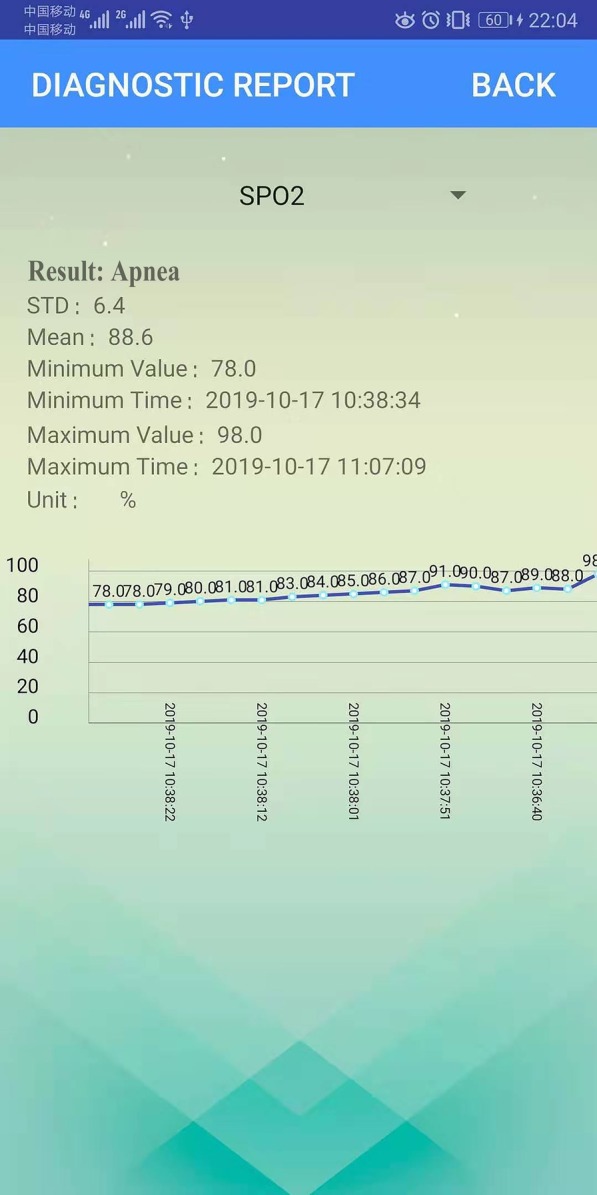
Sleep apnea syndrome report

The principle functions of our system are the storage of the patient physiological data, the preprocessing of the data, and the visualization of the data and results. To achieve this, the Bluetooth module permits a medical device to transmit specific physiological parameters to a smart phone. Next, the smart phone performs a variety of tasks. This includes the adaptive processing of the physiological parameters, the filtering out of noise (e.g. frequency interference or specious signals), and the collection of several physiological parameters including SpO2, breath rate, pulse rate, and End-tidal carbon dioxide (ETCO2). Next, the pulse waveform of ETCO2 and oxygen are displayed in real-time directly on the phone (as shown in Fig. 11). The Bluetooth and mobile phone transmission rate during result display is highly stable at 3.6 KB/s. Resultant data are stored in a cloud database allowing better maintenance. Smart phones can access diagnostic reports located in the cloud at any time, and medical clinicians can monitor patient status remotely. Figure 11 shows the statistical analysis and diagnostic result display of physiological data from 1 min; these include the minimum and maximum values, the mean, and the standard deviation. Furthermore, diagnostic reports can be obtained at any time; examples for non-apnea and apnea patients are shown, respectively, in Figs. 12 and 13.

## Conclusions

OSAS represents a serious breathing disorder that occurs while a patient is sleeping. While the premiere method of OSAS diagnosis is currently PSG, it is both costly and complicated to use. What’s more, PSG requires significant turnaround time for obtaining a diagnosis. In light of this, we have developed a method to detect apnea in real-time. To achieve this, we have developed a system to assess SpO2 statistical features using a SVM classifier, deployed on an IoT-based architecture and cloud computing. This novel methodology confers several advantages over current diagnostic tools. Firstly, cloud computing provides both a low cost, always accessible source of storage and a set of reliable computing resources essential for physiological data analysis. Secondly, our IoT architecture-based sleep monitoring system provides easy to use, remote monitoring facilitating real-time diagnosis. Thirdly, our algorithm can be employed for detecting significant, potentially serious apnea events before consulting with a medical professional for an initial diagnosis. Fourthly, an experimental evaluation of our system’s ability to diagnose OSAS using pre-existing, real-world, clinical data confirm that our algorithm can efficiently diagnose sleep apnea as evidenced by its successfully achieving 87.6%, 90.2%, and 94.1% sensitivity, accuracy, and specificity rates, respectively. Notably, the sensitivity, specificity, and accuracy of our algorithm well surpasses the performance of the existing, widely used DBN and AdaBoost tools.

Of note, in the near future, 5G networks will allow our IoT based application to operate even more efficiently through experiencing less packet loss and higher packet retransmission. In addition to this, we also plan to explore our tool’s utility in classifying apnea severity and for predicting future apnea, both by using the current features and by utilizing additional features, diagnostic record integration, disease histories, and more detailed patient profiles. Moreover, in this study, our method was applied to a small-scale data in which the BMIs of the participants are mostly high. In the future, we will apply it to large-scale data to optimize the performance of our algorithm; we would also like to expand the study to include a range of BMI. Also, we will compare our algorithm with more algorithms and determine the impact on performance as the parameters of the different algorithms change.

## Data Availability

The apnea related data used in this research can be downloaded at: https://physionet.org/content/ucddb/1.0.0/. St. Vincent’s University Hospital / University College Dublin Sleep Apnea Database is an open-access database. This database contains 25 full overnight polysomnograms with simultaneous three-channel Holter ECG, from adult subjects with suspected sleep-disordered breathing.

## Abbreviations

AdaBoost: Adaptive boosting
AHIs: Apnea Hypopnea Indexes
BMIs: Body Mass Indexes
CoAP: Constrained application protocol
DBN: Deep belief network
ECG: Electrocardiography
EEG: Electroencephalography
EMG: Electromyography
EOG: Electrooculography
ETCO2: End-tidal carbon dioxide
FN: False negative
FP: False positive
Hz: Hertz
HTTP: Hypertext transfer protocol
HTTPS: Hypertext transfer protocol secure
IoT: Internet of Things
MQTT: Message queuing telemetry transport
N: Negative
OSAS: Obstructive sleep apnea syndrome
P: Positive
PSG: Polysomnography
QoS: Quality of service
RBF: Radial basis function
SAHS: Sleep apnea–hypopnea syndrome
SAS: Sleep apnea syndrome
SDK: Software development kit
SpO2: Blood oxygen saturation
TCP: Transmission control protocol
TN: True negative
